# A New Prognostic Indicator for Biliary Tract Cancers: The ABIC Score

**DOI:** 10.3390/curroncol32040200

**Published:** 2025-03-28

**Authors:** Doğan Bayram, Öznur Bal, Kemal Karaman, Murat Bardakçı, Derya Demirtaş Esmer, İsmet Seven, Serhat Sekmek, Perihan Perkin, Fahriye Tuğba Köş, Efnan Algın, Doğan Uncu

**Affiliations:** 1 Department of Medical Oncology, Ankara City Hospital, 06800 Ankara, Turkey; drderyademirtas@gmail.com (D.D.E.); sevenismet84@gmail.com (İ.S.); serhatsekmek@gmail.com (S.S.); perihanperkin@gmail.com (P.P.); 2 Department of Medical Oncology, University of Health Sciences, Ankara City Hospital, 06800 Ankara, Turkey; dr_ozn@yahoo.com (Ö.B.); tugbasan@yahoo.com (F.T.K.); efnanalgin@gmail.com (E.A.);; 3 Medical Oncology, Afyonkarahisar State Hospital, 03100 Afyonkarahisar, Turkey; dr.kemal1905md@gmail.com; 4 Diyarbakır Gazi Yaşargil Education Research Hospital, 21010 Diyarbakır, Turkey; dr.muratbardakci@hotmal.com

**Keywords:** biliary tract cancers, ABIC socre, gallbladder cancer, intrahepatic cholangiocarcinoma, extrahepatic cholangiocarcinoma

## Abstract

Introduction: Biliary tract cancers (BTC) comprise a heterogeneous group of malignancies, including gallbladder cancer, intrahepatic cholangiocarcinoma, and extrahepatic cholangiocarcinoma. The main determinants of prognosis in BTC are the stage of the disease and the eligibility for curative treatment. Additionally, liver functional capacity is also one of the factors influencing survival in biliary tract cancers. The age–bilirubin–INR–creatinine (ABIC) score has been previously shown to predict prognosis in hepatic diseases. The aim of our study is to demonstrate the relationship between the ABIC score and prognosis in BTC. Materials and Methods: In this study, a retrospective analysis was performed on 41 patients with non-metastatic BTC and 73 patients with metastatic BTC who were followed up in our clinic between 2003 and 2025. All patients were ≥18 years old at the time of diagnosis, and BTC was pathologically confirmed. The ABIC score was calculated separately for each group. A threshold value for the ABIC score was determined using Receiver Operating Characteristic (ROC) analysis, and based on this threshold, patients were divided into low and high ABIC score groups. Both the relationship between the ABIC score and prognosis and the other factors affecting prognosis were investigated. Results: In the non-metastatic BTC group, the cutoff value for the ABIC score was 6.89. The median survival time of patients with a high ABIC score was significantly shorter. In the metastatic BTC group, the cutoff value for the ABIC score was 7.41. Similarly, in this group, patients with a high ABIC score had a significantly shorter median survival time. Additionally, in the non-metastatic BTC group, tumor localization and stage were prognostic factors affecting survival, while in the metastatic BTC group, CEA and first-line chemotherapy were the prognostic factors influencing overall survival. **Conclusions:** We demonstrate that the ABIC score is a prognostic factor determining median survival in both non-metastatic and metastatic BTC patients.

## 1. Introduction

Biliary tract cancers (BTC) comprise a heterogeneous group of malignancies including gallbladder cancer (GBC), intrahepatic cholangiocarcinoma (IHCC), and extrahepatic cholangiocarcinoma (EHCC) and account for less than 1% of all cancers worldwide [1]. The prognosis is poor, with an all-stage 5-year overall survival of less than 20%. Surgery is the best option for non-metastatic disease because biliary tract cancers (BTCs) are curable when resected [2]. BTCs are rarely diagnosed at an early stage due to the asymptomatic nature of the disease. Therefore, diagnosis typically occurs at an advanced stage. Consequently, curative surgery and adjuvant therapy are only feasible options for a limited subset of patients. Palliative approaches are emphasized for patients who are not suitable for surgery. The prognosis is particularly poor in cases of unresectable or metastatic disease. Standard care for this condition typically involves chemotherapy, radiotherapy, biliary drainage, and stent placement. Despite their rarity, given the overall dismal prognosis associated with BTCs, identifying prognostic and predictive factors seems to be a critical need [3].

Regional differences in cholangiocarcinoma incidence reflect different underlying risk factors. However, conditions that lead to chronic inflammation (primary sclerosing cholangitis, Caroli’s disease, hepatolithiasis, and liver fluke infections cirrhosis, gallstones, hepatitis B and hepatitis C infections, obesity-related liver disease, and diabetes), causing the epithelium to change under stress, trigger the development of cancer [4,5].

The age–bilirubin–international normalized ratio (INR)–creatinine (ABIC) score is based on parameters reflecting age, liver function, coagulation, and kidney function [6]. The ABIC score was initially used to assess survival in alcoholic hepatitis and identify patients who responded to corticosteroid therapy [7]. Considering that age, INR, total bilirubin, and creatinine have been reported as independent risk factors for predicting survival in patients with liver failure, the ABIC score is a guide for determining liver functional capacity [8].

Currently, the ABIC score is used in liver diseases such as alcoholic hepatitis and B virus-related acute-on-chronic liver failure. It is also considered a potential prognostic model for coronary artery disease, where it has been observed that patients with a high ABIC score have a higher rate of cardiac death due to coronary artery disease compared to those with a low ABIC score [9].

There is insufficient literature demonstrating the relationship between the ABIC score and the prognosis of malignant diseases. The aim of our study is to show the relationship between ABIC score and survival in non-metastatic and metastatic biliary tract tumors.

The ABIC score was calculated using the formula ABIC = (age × 0.1) + (serum bilirubin × 0.08) + (serum creatine × 0.3) + (INR × 0.8) [7].

## 2. Material and Methods

### 2.1. Patients and Study Design

In this study, a retrospective analysis was performed on 41 non-metastatic and 73 metastatic patients with biliary tract cancer who were followed up in our clinic between 2003 and 2025. All patients were ≥18 years old at the time of diagnosis, and BTC was pathologically confirmed. Clinicopathological features, as well as hematological and biochemical results, of the patients were obtained from the hospital registration system and reviewed retrospectively. Patients with missing data were excluded. Staging was determined based on the eighth edition of the American Joint Committee on Cancer (AJCC) Staging Manual [10]. Tumor response was assessed using computerized tomography and in accordance with the RECIST criteria [11].

The patients in this study were divided into two separate groups: non-metastatic (stage I/II/III) and metastatic (stage IV) patients. The ABIC score was calculated separately for each group using the formula: (age × 0.1) + (serum bilirubin × 0.08) + (serum creatinine × 0.3) + (INR × 0.8) [7]. A threshold value for the ABIC score was determined using Receiver Operating Characteristic (ROC) analysis, and based on this threshold, patients were divided into low and high ABIC score groups.

Overall survival (OS) was defined as the time interval from the onset of the disease to death from any cause or the last follow-up. Serum total bilirubin >1.2 mg/dL was defined as jaundice. Elevated liver transaminases, aspartate aminotransferase (AST), and alanine aminotransferase (ALT) were defined as AST and ALT > 40 U/L. The upper limit for alkaline phosphatase (ALP) was set at 130 IU/mL, and for Gamma-glutamyl transferase (GGT), it was 40 U/L. Carcinoembryonic Antigen (CEA) > 5 ng/mL and Carbohydrate antigen 19-9 (CA19-9) > 30 U/mL were considered the upper limits of normal.

Biliary tract cancer (BTC) patients were categorized into two groups based on tumor localization: gallbladder cancer and intrahepatic/extrahepatic bile duct cancer. Median age was calculated separately for both non-metastatic and metastatic groups, and patients in each group were further divided into two subgroups based on whether their age was below or above the median. Performance status was classified into two groups: ECOG 0–1 and ECOG 2–4. Smoking status was categorized into two groups: current and former smokers were grouped together, while never-smokers formed a separate group. Patients were also stratified based on their CEA, CA 19-9, ALT, AST, GGT, ALP, and total bilirubin levels as either normal or elevated. Survival analyses included assessments of age, sex, comorbidities, tumor localization, ECOG performance status, smoking history, CEA, CA 19-9, ALT, AST, GGT, ALP, total bilirubin levels, and ABIC score.

### 2.2. Statistical Analyses

Statistical analysis was performed using the Statistical Package for the Social Sciences Version 22.0 for Windows (SPSS et al., Chicago, IL, USA). Two groups were compared with the Mann–Whitney U test and Pearson Chi-square or Fisher tests for continuous and categorical variables, respectively. Survival analysis was carried out by the Kaplan–Meier method using the Log-rank test. Survival times were determined within a 95% CI (Confidence Interval) range. *p* < 0.05 was considered statistically significant. The cutoff point with best sensitivity and specificity of the new score was determined through the receiver-operating characteristic curve (ROC) coordinates.

## 3. Results

### 3.1. Patient Characteristics

The patients were divided into two groups: non-metastatic and metastatic.

**Figure 1:**
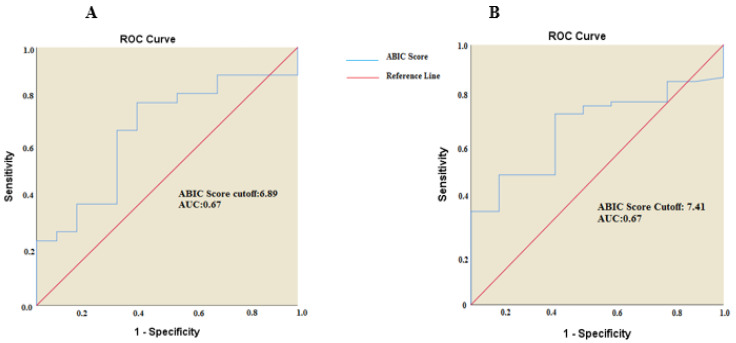
ABIC score cutoff values. (**A**). Non-metastatic BTC group. (**B**). Metastatic BTC group.

The non-metastatic group comprised 41 patients with a median age of 62 years (range: 30–78). Twenty-one patients (51.2%) were aged 62 years or younger, while 20 patients (48.8%) were older than 62 years. Of these patients, 73.2% were female and 26.8% were male. Tumor localization was in the gallbladder in 75.6% of cases, while 24.4% had intrahepatic or extrahepatic bile duct cancer. A history of smoking was present in 39% of patients, and 56.1% had at least one comorbidity. Regarding performance status, 73.2% of patients had an ECOG score of 0–1, whereas 26.8% had an ECOG score of 2–4. Elevated CEA levels were observed in 14.6% of patients, elevated CA 19-9 in 2.8%, elevated GGT in 53.7%, elevated total bilirubin in 14.6%, elevated ALP in 22%, elevated ALT in 12.2%, and elevated AST in 22%. Based on ROC analysis, the cutoff value for the ABIC score in this group was determined to be 6.89 (AUC: 0.676). Among the patients, 31.7% had an ABIC score below 6.89, while 68.3% had a score above this threshold (Figure 1).

The metastatic group consisted of 73 patients, with a median age of 63 (36–82). Thirty-six patients (49.3%) were aged 64 years or younger, while 37 patients (50.7%) were older than 64 years. Among the patients, 43.8% were female and 56.2% were male. The primary tumor was localized in the gallbladder in 52.1% of cases, while 47.9% had intrahepatic or extrahepatic bile duct cancer. A history of smoking was present in 46.6% of patients, and 53.4% had at least one comorbidity. Regarding performance status, 67.1% of patients had an ECOG score of 0–1, whereas 32.9% had an ECOG score of 2–4. Elevated CEA levels were observed in 41.1% of patients, elevated CA 19-9 in 80.8%, elevated GGT in 91.8%, elevated total bilirubin in 46.6%, elevated ALP in 63%, elevated ALT in 53.4%, and elevated AST in 54.8%. Based on ROC analysis, the cutoff value for the ABIC score in this group was determined to be 7.41 (AUC: 0.673). Among the patients, 31.5% had an ABIC score below 7.41, while 68.5% had a score above this threshold (Figure 1).

The demographic and clinical characteristics of the patients are summarized in Table 1.

### 3.2. Treatment Modalities

In our study, all patients in the non-metastatic group underwent curative surgery. Three patients (7.3%) received neoadjuvant chemotherapy, while 28 patients (68.2%) received adjuvant chemotherapy. The neoadjuvant chemotherapy regimen for all three patients was gemcitabine and cisplatin. The most commonly administered adjuvant chemotherapy regimen was single-agent gemcitabine (28.6%), followed by gemcitabine-cisplatin (21.4%) and FOLFOX (21.4%). Adjuvant chemoradiotherapy (CRT) was applied to 13 patients (31.7%). Concurrent chemotherapy with radiotherapy included 5-FU in ten patients, gemcitabine in two patients, and capecitabine in one patient. Recurrence occurred in 21 patients (51.2%) in the non-metastatic group. Of the recurrences, 5 were local relapses, and 16 were distant metastases. First-line chemotherapy was administered to 14 patients (34.1%) in the non-metastatic group. The most frequently used chemotherapy regimen in the first line was gemcitabine-cisplatin (50%), followed by single-agent gemcitabine (21.4%), FOLFOX (14.2%), cisplatin-5FU (7.1%), and capecitabine (7.1%). Progression was observed in 12 patients after first-line chemotherapy. Three patients received second-line chemotherapy. For second-line chemotherapy, two patients (66.7%) received gemcitabine-carboplatin, and one patient (33.3%) received gemcitabine-cisplatin.

Of the 73 patients in the metastatic group, 57 (78.1%) received first-line chemotherapy. Sixteen patients (21.9%) did not receive chemotherapy and were managed with palliative supportive care. The most commonly used first-line chemotherapy regimen was gemcitabine-cisplatin (45.6%), followed by single-agent gemcitabine (22.8%), FOLFOX (12.3%), cisplatin-5FU (10.5%), gemcitabine-capecitabine (3.5%), single-agent capecitabine (3.5%), and gemcitabine-oxaliplatin (1.8%). Progression was observed in 51 patients who received first-line chemotherapy. Second-line chemotherapy was administered to 11 patients (15%). The most frequently used second-line regimen was gemcitabine-cisplatin (36.4%). Second-line regimens included FOLFOX (27.2%), gemcitabine-carboplatin (18.2%), and single-agent capecitabine (18.2%). The treatment modalities of the patients are shown in Table 2.

### 3.3. Survival Analysis and Treatment Effect

**Figure 2:**
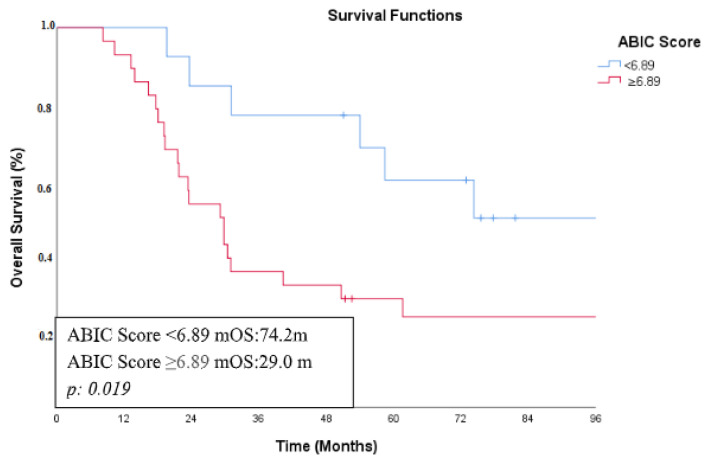
Kaplan–Meier curve showing overall survival based on ABIC score in non-metastatic BTC group.

The median follow-up time of the 41 patients in the non-metastatic group was 120.8 (95% CI: 50.8–190.8) months, and the median overall survival time was 31.08 (95% CI: 4.86–57.29) months. In the non-metastatic patient group, no statistically significant association was found between overall survival and sex, age, smoking history, comorbidities, ECOG performance status, adjuvant chemotherapy, adjuvant chemoradiotherapy, or elevated levels of CEA, CA 19-9, ALT, AST, GGT, and ALP. In univariate analysis, primary tumor location, stage, and ABIC score were found to be prognostic factors that could affect overall survival. Among patients with gallbladder tumors, the median overall survival (mOS) was 53.9 months, whereas it was 18 months in those with intrahepatic or extrahepatic bile duct cancer. Patients with stage 1–2 disease had an mOS of 58.4 months, while those with stage 3 disease had an mOS of 21.7 months. Regarding the primary objective of this study, which was to assess the impact of the ABIC score on survival, patients with an ABIC score below 6.89 had an mOS of 74.2 months, whereas those with a score of 6.89 or higher had an mOS of 29 months (Figure 2). In multivariate analysis using Cox regression, tumor location (*p*: 0.012, HR: 0.320, 95% CI: 0.13–0.77), stage (*p*: 0.009, HR: 0.322, 95% CI: 0.13–0.75), and ABIC score (*p*: 0.031, HR: 0.361, 95% CI: 0.14−0.91) were determined to be independent prognostic factors affecting overall survival. The survival analysis results for the non-metastatic patient group are shown in Table 3.

The median follow-up time of the 73 patients in the metastatic group was 158.1 months, and the median overall survival time was 7.75 (95% CI: 6.15−9.35) months. In the metastatic patient group, no statistically significant association was found between overall survival and sex, age, smoking history, comorbidities, primary tumor localization, or elevated levels of CA 19-9, ALT, AST, GGT, and ALP.

**Figure 3:**
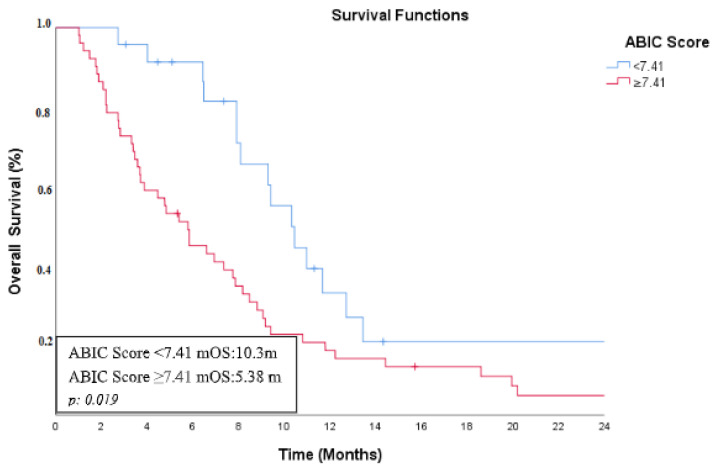
Kaplan–Meier curve showing overall survival based on ABIC score in metastatic BTC group.

In the univariate analysis, ABIC score, CEA levels, ECOG performance status, and receipt of first-line chemotherapy were found to be significant prognostic factors for overall survival. Patients with an ABIC score below 7.4 had an mOS of 10.3 months, whereas those with a score of 7.41 or higher had an mOS of 5.38 months (Figure 3). Similarly, patients with normal CEA levels had an mOS of 7.91 months, compared to 3.87 months in those with elevated CEA levels. The mOS was 8.18 months in patients with an ECOG performance status of 0–1, whereas it was 3.71 months in those with an ECOG score of 2–4. Additionally, patients who received first-line chemotherapy had an mOS of 8.08 months, while those who did not receive chemotherapy had an mOS of 3.8 months.

In multivariate analysis using Cox regression, ABIC score (*p*: 0.021, HR: 0.515, 95% CI: 0.29−0.90), elevated CEA (*p*: 0.022, HR: 0.547, 95% CI: 0.32−0.91), and first-line chemotherapy (*p*: 0.001, HR: 0.283, 95% CI: 0.15−0.53) were determined to be independent prognostic factors affecting overall survival. The survival analysis results for the metastatic patient group are presented in Table 4.

## 4. Discussion

In this study, we aimed to demonstrate the relationship between ABIC score and prognosis in biliary tract tumors. A high ABIC score was associated with poor prognosis in both non-metastatic and metastatic biliary tract tumors. Furthermore, in the non-metastatic group, intra-extrahepatic biliary tract tumors had a worse prognosis compared to gallbladder tumors, and stage 3 tumors had a worse prognosis compared to stage 1−2 tumors. In the metastatic group, patients with elevated CEA and those who did not receive first-line chemotherapy had a worse prognosis. In our study, the cutoff value for the ABIC score was found to be 6.89 for the non-metastatic group and 7.41 for the metastatic group. There is no universally accepted cutoff value for the ABIC score in the literature.

The ABIC score may serve as a valuable prognostic tool in biliary tract cancer (BTC) by reflecting both hepatic dysfunction and systemic disease burden. BTC is an aggressive malignancy characterized by early metastasis, biliary obstruction, and progressive liver dysfunction, all of which contribute to a deterioration in biochemical and clinical parameters. Hyperbilirubinemia is directly associated with biliary obstruction and tumor burden, while elevated creatinine may indicate systemic inflammation, sepsis, or chemotherapy-induced nephrotoxicity—common complications in advanced BTC. Additionally, prolonged INR reflects impaired hepatic synthetic function, often exacerbated by tumor progression and underlying liver disease. The inclusion of age further strengthens the prognostic value of the ABIC score, as elderly BTC patients tend to be more frail and have lower treatment tolerance. Given that BTC frequently leads to rapid deterioration in liver function and systemic status, the ABIC score provides a comprehensive assessment of disease severity and may play a crucial role in risk stratification, treatment decision-making, and survival prediction in this patient population.

In the study by Dominguez et al., the contribution of the ABIC score in determining the risk of death due to alcoholic hepatitis was investigated. The ABIC score was categorized as low (<6.71), intermediate (6.71−9), or high (>9). The 90-day survival rate was 100% in the low ABIC score group, while it was 25% in the high ABIC score group [7].

In the study by Jia et al., a high ABIC score was shown to be a positive predictor in patients with alcohol-associated liver carcinoma. In that study, the cutoff value for the ABIC score was found to be 6.99. In the group of patients with alcohol-associated liver diseases but without liver carcinoma, the ABIC score was 6.96, while in the group with alcohol-associated liver diseases who developed liver carcinoma, the ABIC score was 7.74 (*p* < 0.001). It was stated that the ABIC score has diagnostic value for alcohol-associated liver carcinoma [12].

In the study by Chen et al., the predictive value of the ABIC score was investigated in patients with acute-on-chronic hepatitis B liver failure. It was found that patients with an ABIC score above 9.44 had higher mortality rates. Additionally, the ABIC score was shown to be superior to the Model for End-stage Liver Disease (MELD) score in predicting short-term survival [13].

Wu et al. examined the relationship between the ABIC score and prognosis in patients with coronary artery disease. In this study, the cutoff value for the ABIC score was determined to be 7.98. Among patients who underwent percutaneous coronary intervention, those with a high ABIC score had higher long-term mortality rates. In the high ABIC group, all-cause mortality was 1.7 times higher (*p* < 0.001), and cardiac-related mortality was 1.5 times higher (*p* = 0.005) [9].

In our study, we also demonstrated that the ABIC score has prognostic value in both non-metastatic and metastatic BTC. This can primarily be attributed to the components of the ABIC score (age, bilirubin, INR, creatinine), which are prognostic factors in BTC. Age is a well-known prognostic factor for various tumor types, and five-year survival rates are significantly higher in younger patients compared to older ones [14]. The inclusion of bilirubin as a parameter is noteworthy, as elevated Albumin-Bilirubin (ALBI) scores are associated with poor prognosis in both non-metastatic and metastatic BTC cases [15,16]. Elevated creatinine levels in patients often indicate malnutrition and poor treatment tolerance, which negatively impacts prognosis. These patients are typically unable to receive cisplatin-based chemotherapy regimens. Similarly, Subbiah et al.’s study found that elevated creatinine levels were associated with shorter PFS durations in biliary tract cancer [17]. The INR level is particularly important for indicating post-hepatectomy liver failure in BTC patients who undergo surgery. As a parameter of the Child–Turcotte–Pugh (CTP) score, INR is relevant since patients classified as CTP stages B and C have a higher risk of post-hepatectomy liver failure compared to those in stage A [18].

The reason why an elevated ABIC score, which includes age, bilirubin, creatinine, and INR parameters, is a poor prognostic factor in biliary tract cancers is that each of these parameters individually has a significant impact on the clinical course of cancer patients. Since these parameters reflect severe systemic conditions such as liver dysfunction, renal failure, and coagulopathy, an increased ABIC score is associated with worse overall survival. In our study, the presence of additional comorbidities, a history of smoking, and a higher ECOG performance score were more prevalent in patients with a high ABIC score in both the non-metastatic and metastatic groups compared to those with a low ABIC score. All of these adverse characteristics are poor risk factors for BTC patients and contribute to deterioration in both clinical and laboratory parameters, ultimately leading to a shorter patient survival. Another important point is that patients with a high ABIC score were less likely to receive effective treatment. In particular, in the metastatic group, the proportion of patients managed with the best supportive care was higher among those with a high ABIC score compared to those with a low ABIC score. In biliary tract cancers, cisplatin-based treatments, which form the cornerstone of therapy, are often contraindicated in patients with elevated creatinine and bilirubin levels due to their potential nephrotoxic and hepatotoxic effects, further limiting treatment options for these high-risk patients

The MELD score was initially developed to assess the three-month mortality risk in patients with liver cirrhosis and to prioritize candidates for liver transplantation [19]. The Child–Pugh (CP) score remains the most commonly used tool for evaluating liver function. However, its reliance on subjective parameters, such as ascites and hepatic encephalopathy, presents certain limitations. In recent years, the albumin-to-bilirubin (ALBI) score, which consists of only two serum parameters, has been introduced as a prognostic indicator for hepatocellular carcinoma (HCC) and has proven useful in stratifying patients within CP class A [20].

Originally developed for the assessment of alcoholic hepatitis, the ABIC score has recently been investigated as a prognostic marker in alcohol-associated liver carcinoma and acute-on-chronic hepatitis B liver failure. Unlike the MELD and Child–Pugh scores, which are primarily used to evaluate liver cirrhosis and prioritize liver transplantation candidates, the ABIC score incorporates age in addition to bilirubin, creatinine, and INR. This inclusion makes the score a more comprehensive measure that better reflects overall patient frailty.

While the MELD and ALBI scores rely solely on objective biochemical parameters, the Child–Pugh score includes subjective assessments such as ascites and hepatic encephalopathy, which may introduce interobserver variability. In contrast, the ALBI score was specifically designed to classify hepatocellular carcinoma (HCC) patients, particularly those within Child–Pugh class A, based solely on albumin and bilirubin levels. Given these distinctions, the ABIC score may offer prognostic value in BTC patients by integrating both hepatic and systemic parameters to assess disease severity and survival outcomes.

The limitations of our study include its retrospective design and the small number of patients. Further studies are needed to better elucidate the relationship between the ABIC score and cancer prognosis.

## 5. Conclusions

Our study demonstrated that a high ABIC score is a poor prognostic factor in both non-metastatic and metastatic BTC patients. Median survival times were lower in patients with high ABIC scores in both groups. Additionally, tumor localization and stage were prognostic factors in the non-metastatic BTC group, while CEA levels and first-line chemotherapy were significant prognostic factors in the metastatic BTC group. These findings suggest that the ABIC score may serve as a prognostic indicator in other cancer types as well.

## Figures and Tables

**Table 1:** Demographic and clinical characteristics of the patients.

		Non-Metastatic Group		Metastatic Group	
		n	(%)	n	(%)
Number of Patients		41		73	
Median Age (min–max)		62 (30–78)		63 (36–82)	
Gender	Female	30	(73.2)	32	(43.8)
	Male	11	(26.8)	41	(56.2)
Location	GBC	31	(75.6)	38	(52.1)
	IHCC and EHCC	10	(24.4)	35	(47.9)
Stage at diagnosis	I	4	(9.8)		
	II	23	(56.1)		
	III	14	(34.1)		
	IV	0	(0)	73	(100)
Comorbidity	Yes	23	(56.1)	39	(53.4)
	No	18	(53.9)	34	(46.6)
Smoking History	Yes (former smoker or current smoker)	16	(39)	34	(46.6)
	No	25	(61)	39	(53.4)
CEA	Normal	35	(85.4)	43	(58.9)
	Elevated	6	(14.6)	30	(41.1)
CA 19-9	Normal	30	(73.2)	14	(19.2)
	Elevated	11	(2.8)	59	(80.8)
GGT	Normal	19	(46.3)	6	(8.2)
	Elevated	22	(53.7)	67	(91.8)
T. Bilirubin	Normal	35	(85.4)	39	(53.4)
	Elevated	6	(14.6)	34	(46.6)
ALP	Normal	32	(78)	27	(37)
	Elevated	9	(22)	46	(63)
ALT	Normal	36	(87.8)	34	(46.6)
	Elevated	5	(12.2)	39	(53.4)
AST	Normal	32	(78)	33	(45.2)
	Elevated	9	(22)	40	(54.8)
ECOG	0−1	30	(73.2)	49	(67.1)
	2−4	11	(26.8)	24	(32.9)
ABIC Score (ROC Analysis)		6.89		7.41	
ABIC Score	Low	13	(31.7)	23	(31.5)
	High	28	(68.3)	50	(68.5)

n: number, IHCC: intrahepatic cholangiocarcinoma, EHCC: extrahepatic cholangiocarcinoma, ECOG: Eastern Cooperative Oncology Group, min: minimum; max: maximum. ALP: alkaline phosphatase, GGT: Gamma-glutamyl transferase, CEA: Carcinoembryonic Antigen, CA19-9:Carbohydrate antigen 19-9, ABIC: age–bilirubin–INR–creatinine, ALT: Alanine Aminotransferase, AST: Aspartate Aminotransferase.

**Table 2:** Treatment modalities of patients.

		Non-Metastatic Group		Metastatic Group	
		n: 41	(%)	n: 73	(%)
Curative Surgery	Yes	41	(100)	0	(0)
	No	0	(0)	73	(100)
Neoadjuvant CT	Gemcitabine-Cisplatin	3	(7.31)	0	(0)
Adjuvant CT		28	(68.2)	0	(0)
	Gemcitabine	8	(28.6)		
	Gemcitabine-Cisplatin	6	(21.4)		
	Gemcitabine-Capecitabine	3	(10.7)		
	FOLFOX	6	(21.4)		
	Cisplatin-5 Fluorouracil	4	(14.3)		
	Capecitabine	1	(3.6)		
Adjuvant CRT		13	(31.7)	0	(0)
Relapse		21	(51.2)		
First-Line CT	Yes	14	(34.1)	57	(78.1)
First-Line CT Regimen	Gemcitabine-Cisplatin	7	(50)	26	(45.6)
	Gemcitabine	3	(21.4)	13	(22.8)
	FOLFOX	2	(14.2)	7	(12.3)
	Cisplatin-5 Fluorouracil	1	(7.1)	6	(10.5)
	Gemcitabine-Capecitabine	0		2	(3.5)
	Capecitabine	1	(7.1)	2	(3.5)
	Gemcitabine-Oxaliplatin	0		1	(1.8)
Progressive disease after first-line CT		12	(29.2)	51	(69.9)
Second-Line CT	Yes	3	(7.3)	11	(15)
Second-Line CT Regimen	Gemcitabine-Cisplatin	1	(33.3)	4	(36.4)
	Gemcitabine-Carboplatin	2	(66.7)	2	(18.2)
	FOLFOX	0		3	(27.2)
	Capecitabine	0		2	(18.2)

n: number, CT: Chemoteharpy.

**Table 3:** Univariate and multivariate analysis of factors affecting overall survival in the non-metastatic BTC group.

		Median OS (Months)	Univariate (*p* Value)	Multivariate *p* Value	HR
Gender	Female	31.08	0.786		
	Male	21.48			
Age Group	≤62	53.9	0.116		
	>62	23.5			
Comorbidity	Yes	30.3	0.193		
	No	40.3			
Smoking	Yes (former smoker or current smoker)	29.0	0.650		
	No	40.3			
Location	GBC	53.9	0.013	0.012	0.320
	IHCC and EHCC	18.0			
Stage	I–II	58.4	0.012	0.009	0.322
	III	21.7			
ABIC Score	<6.89	74.2	0.019	0.031	0.361
	≥6.89	29.0			
CEA	Normal	50.6	0.146		
	Elevated	17.6			
CA 19-9	Normal	31.0	0.542		
	Elevated	19.2			
AST	Normal	31.0	0.144		
	Elevated	29.7			
GGT	Normal	58.4	0.432		
	Elevated	29.73			
ALT	Normal	50.6	0.750		
	Elevated	30.3			
ALP	Normal	61.5	0.622		
	Elevated	30.3			
ECOG	0−1	31.6	0.054		
	2−4	30.3			
Adjuvant CT	Yes	58.4	0.138		
	No	29.7			
Adjuvant CRT	Yes	30.9	0.478		
	No	40.3			

n: number, ECOG: Eastern Cooperative Oncology Group, ALP: alkaline phosphatase, GGT: Gamma-glutamyl transferase, CEA: Carcinoembryonic Antigen, CA 19-9:Carbohydrate antigen 19-9, ABIC: age-bilirubin-INR-creatinine, ALT: Alanine Aminotransferase, AST: Aspartate Aminotransferase, CT: Chemoteharpy, CRT: Chemoradiotherapy, OS: overall survival, HR: Hazard Ratio, IHCC: intrahepatic cholangiocarcinoma, EHCC: extrahepatic cholangiocarcinoma.

**Table 4:** Univariate and multivariate analysis of factors affecting overall survival in the metastatic BTC group.

		Median OS (Months)	Univariate (*p* Value)	Multivariate *p* Value	HR
Gender	Female				
	Male				
Age Group	≤64	7.91	0.985		
	>64	7.35			
Comorbidity	Yes	7.85	0.60		
	No	6.93			
Smoking	Yes (former smoker or current smoker)	6.43	0.294		
	No	7.85			
Location	GBC	7.91	0.485		
	IHCC and EHCC	5.78			
ABIC Score	<7.41	10.3	0.019	0.021	0.515
	≥7.41	5.38			
CEA	Normal	7.91	0.038	0.022	0.547
	Elevated	3.87			
CA19.9	Normal	8.18	0.207		
	Elevated	6.93			
AST	Normal	8.18	0.245		
	Elevated	4.76			
ALT	Normal	7.91	0.533		
	Elevated	6.93			
GGT	Normal	10.3	0.142		
	Elevated	6.93			
ALP	Normal	7.91	0.407		
	Elevated	7.35			
ECOG	0−1	8.18	0.002	0.519	
	2−4	3.71			
First-Line CT	Yes	8.08	0.001	0.001	0.283
	No	3.38			

## Data Availability

The data presented in this study are available on request from the corresponding author.
